# Hand Skin Burn as a Complication of Electrosurgery Use in Prone Position in Surgery: A Case Report

**DOI:** 10.7759/cureus.10101

**Published:** 2020-08-28

**Authors:** Saud A Sultan, Bassam Alahmadi, Abdullah Mohabbat

**Affiliations:** 1 Orthopedics, King Fahad Hospital, Madina, SAU

**Keywords:** electrosurgery, electrocautery, burns, prone position, orthopedics, spine, surgery, complications, surgical equipment, iatrogenic

## Abstract

Electrosurgery is one of the advances in the surgical field and used commonly. Modern electrosurgical units considered relatively safe. Although rare, inadvertent skin burns due to electrosurgery caused by different mechanisms were reported in the literature. Positioning the patient in prone is required for some surgeries and utilizing this position in a proper manner is essential to avoid complications. We present a case of a 47-year-old female patient who underwent uneventful spinal surgery in a prone position. The patient complained of pain in fingers postoperatively that revealed third-degree skin burn. Plastic surgery was involved in the treatment of burns and the patient followed regularly until fully healed. This case study aims to prompt awareness among surgeons and the staff of the operation-room regarding the unintended burn of patients caused by aberrant circuit related to electrosurgery in prone position.

## Introduction

Electricity is an energy form that has been utilized extensively in all aspects of our lives, including the surgical field. Since the 1920s, Bovie has developed the electrosurgical unit for use in the operation room for cutting and dissecting tissues [1]. The development of this invaluable device has ensued ever since increasing its efficiency and safety. Electrosurgical unit (ESU) delivers electrical current to the soft tissue, and resistance converts it to the desired thermal effect on target tissues. The electrical current travels in a circuit which is composed of a generator that produces the current, an active electrode that delivers it to patient’s body, and a neutral electrode that returns the electrical current which is either away from the active electrode (i.e., a dispersive pad) and named monopolar system, or near the positive electrodes and named bipolar system [2, 3]. ESU is of paramount importance in nowadays-surgical rooms and is utilized efficiently in spine surgeries. Its use is relatively safe but still has the potential for complications. Complications range from tripping on wires to combustion and burns [4]. The burn is a potential hazard that can harm patients or operation room (OR) staff. Burns related to electrosurgery can occur as a result of direct burn by inappropriate activation, heating of pooled liquids, improper contact at dispersive pad site, and aberrant circuit [5-8]. Understanding the principals and knowing the potential complications of electrosurgery in addition to the basic training of using these devices might lower the risk of inflecting harm [9].

The choice of patient position in OR is based on the surgeon and dictated by the condition of the patient and the required surgical procedure, but positioning the patient optimally in a safe manner is a shared responsibility of surgeon, anesthesiologist, and OR nurses. Complications related to surgical positioning are reported in the literature [10]. The prone position is needed in back surgeries as in the spine. It has a unique set of complications that should be known to surgeons and OR staff as it could have the potential of inflecting morbidity to patients. Complications could affect anywhere from forehead down to toes. Although the occurrence of these complications is not uncommon, some are rare and include visual loss and pressure ulcers [11, 12].

Patients safety is a priority and should be ensured by all OR staff as a team. Complications due to electrosurgery and prone positioning could happen. Knowledge about these complications is essential as they are predictable; hence, preventable.

## Case presentation

A 46-year-old female, right-handed, house-wife, not known to have any chronic medical illnesses before, was admitted through the outpatient department with L5-S1 spondylolistheses grade 2 with bilateral severe foraminal narrowing that has failed conservative treatment and was increasing with progressive radiculopathy to both lower limbs. She was booked for elective wide decompression and L5-S1 instrumentation and in situ fusion. Of note, thorough preoperative assessment in the clinic upon admission was conducted with unremarkable findings other than those related to the spine diagnosis.

The patient was successfully shifted to OR designated for spine surgeries. She was placed in a prone position on the Jackson table after being successfully and uneventfully anesthetized and intubated. Her head rested on a gel prone-headrest. Other bony prominences were carefully protected. Both arms were positioned in 90 degrees flexion of shoulder and elbow. They were both placed on well-padded arm boards beside and parallel to the table. The level of both hands was slightly cephalic to the top of the cranium. The grounding pad of the electrosurgical unit was placed in the left thigh. A screen made by the drapes was supported by two intravenous (IV) metallic poles on either side of the patients at the hands level. Screen separated the surgical field from the anesthesia team. The operation underwent smoothly and lasted for one and a half hours. A monopolar system has been used throughout the surgery. The patient has been successfully awakened and extubated and shifted uneventfully to the recovery area. In the recovery area, the pain at the surgical site was controlled, but the patient complained of discomfort in fingers at her right hand; she informed the recovery nurse and orthopedic resident about it but was unchecked as thought to be disoriented after anesthesia.

**Figure 1:**
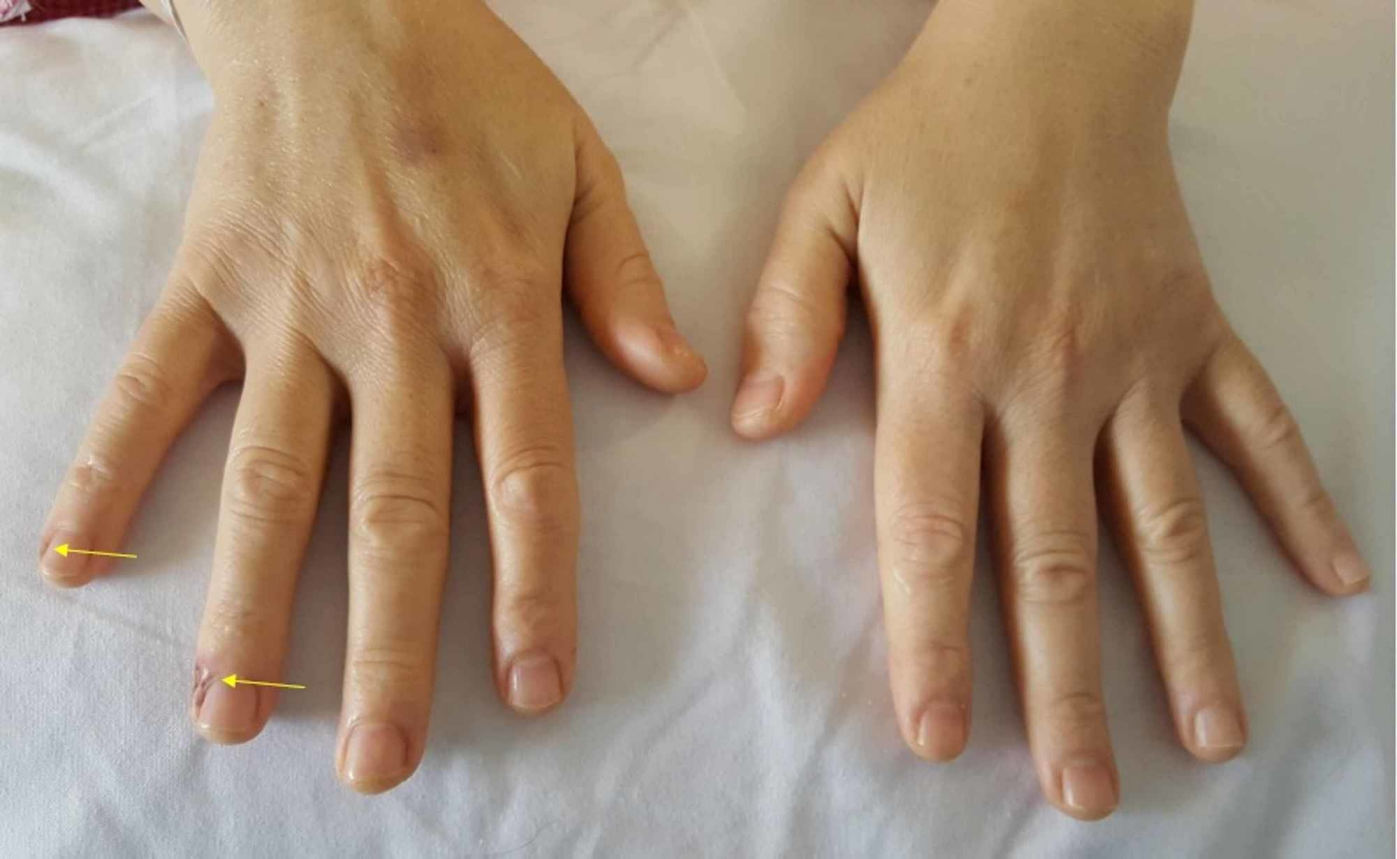
First day after surgery Showing burns on the right fourth and fifth fingers on the ulnar aspect distal to DIPJ. DIPJ - distal interphalangeal joint

**Figure 2:**
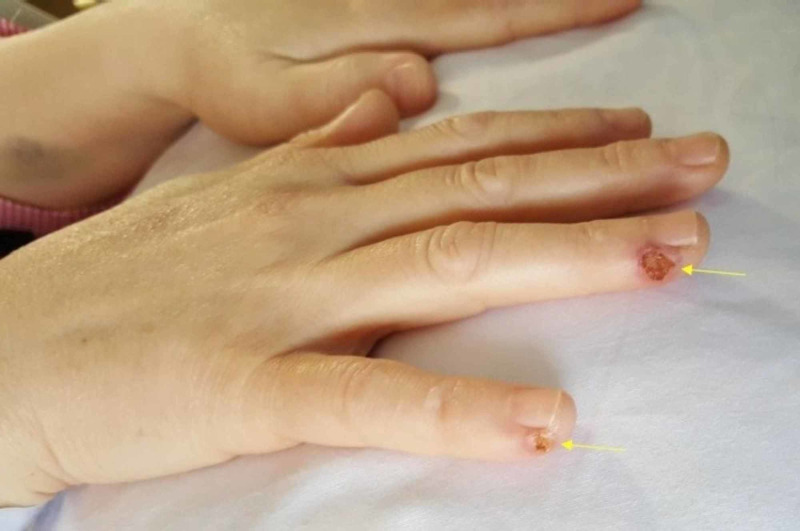
Burns on the right hand first day after surgery

In the ward, after three hours of surgery, the patient was checked upon by the surgical team - she was stable and doing fine. She brought attention again to the pain at her right fingers in addition to noticing skin lesions. Her complaint was inspected this time. There were two deep ulcers on the ulnar aspect of fourth and fifth fingers, approximately 1 cm distal to the distal interphalangeal joint (DIPJ). The ulcers were dry, had red tender edges, deep but not reaching to the bone and had burned base. The size of the fourth finger’s ulcer was about 0.7 x 0.7 cm, and the fifth finger size was about 0.5 x 0.5 cm. Distal sensation remained intact, and a capillary refill was within two seconds. Movements of the fingers, especially at DIPJ, produced pain at the ulcer site. Photos were taken on the first day postop (Figures 1, 2).

**Figure 3:**
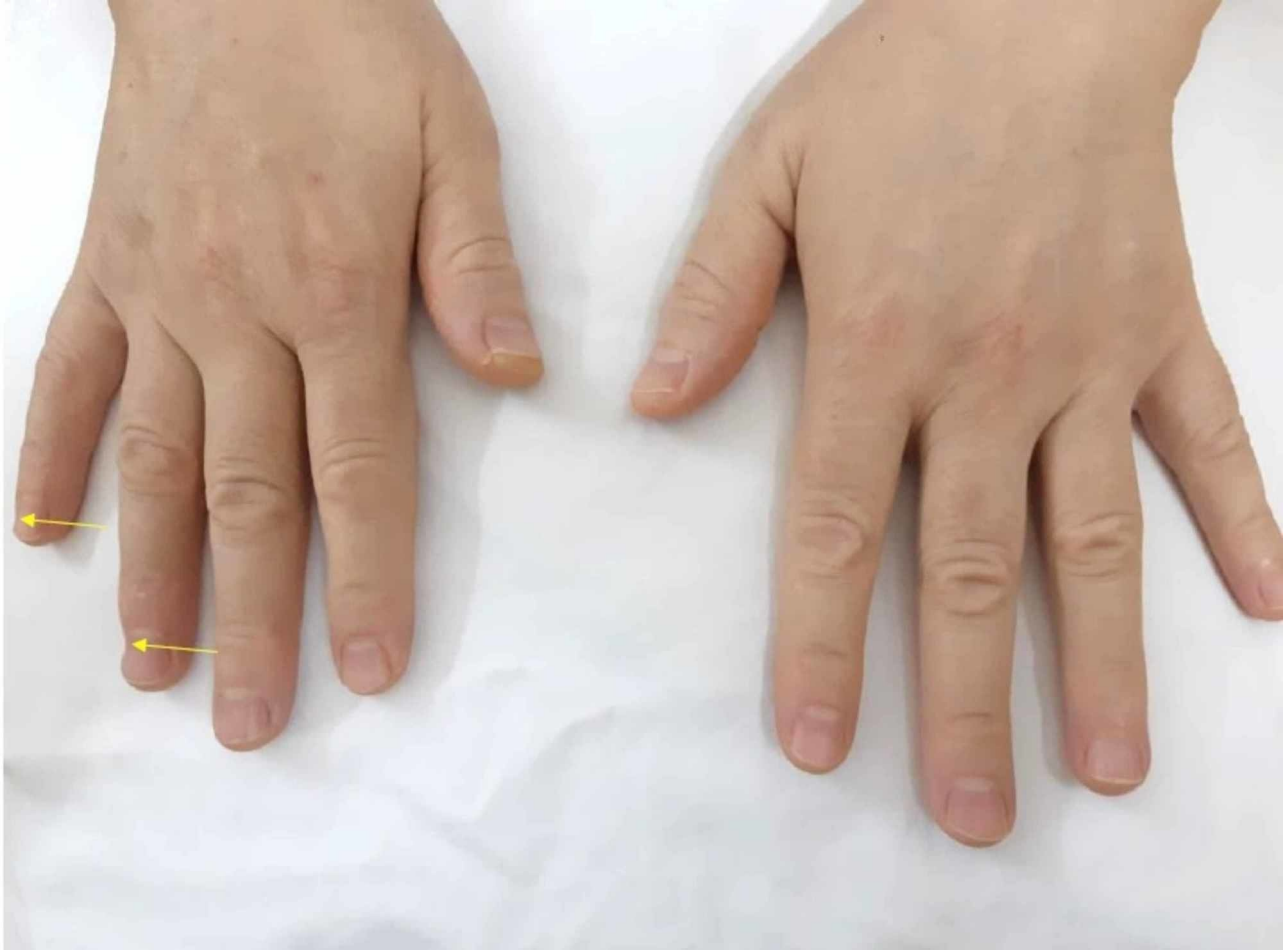
Two years after surgery Showing right fourth and fifth fingers soft tissue defect.

**Figure 4:**
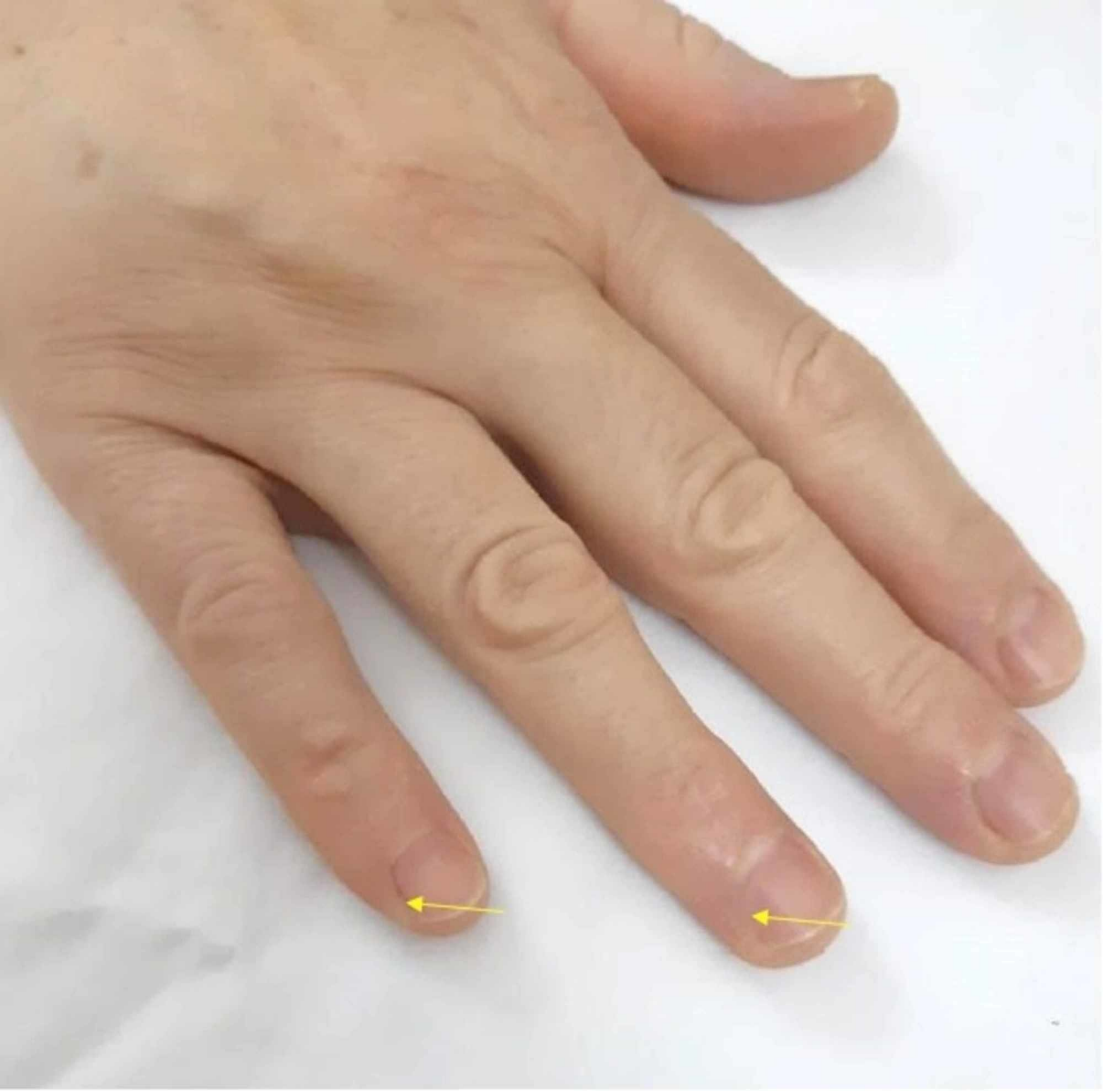
Healed burns on the right hand two years after surgery

It was consulted with plastic surgery, and the diagnosis of third-degree skin burn was confirmed. An incident report was filed. The burns were treated conservatively with daily dressing using silver sulfadiazine cream and the patient was followed by a plastic team regularly until fully healed. Ultimately, no surgical intervention was required for the burns. Hospital stay prolonged for five days more than the expected date of discharge. The surgical team kept a high vigilance for any new burn injuries during her hospital stay. During follow-up, the patient stated that she faced some pain initially when using her right hand for the daily activities and then subsided to discomfort until asymptomatic about four months postop. The patient has been regularly following-up with spine surgery for two years in which her spine problem has improved. The last visit revealed full healing at the burnside, but the residual defect is present with no functional compromise (Figures 3, 4).

## Discussion

The electrosurgical unit is an important part of modern OR. Incorporating the patient’s body in the electric circuit as in monopolar mode is not hazard-free [2, 8]. Despite the great advances in the safety of ESU, complications and injuries to patients and OR staff due to ESU still do occur [2, 3]. Injuries related to ESU use are reported in the literature and roughly estimated to be one-two per 1000 cases. Although being rare, inadvertent burns have no clear incidence, but they are ominous ones [2, 4]. Mundinger et al. discussed the mechanisms of skin burn that can be sustained intraoperatively by ESU and divided them as follow: direct contact burns as a result of inappropriate use of an active electrode, burns at the grounding electrode site due to improper attachment, burns as a result of heating of pooled solutions caused by active or neutral electrodes, and burns occurring outside the operative field as a result of circuits generated between the active electrode and an alternate grounding source [7, 13].

Alternative site burns occur when the patient’s skin is in contact with conductive materials, and the electric currents return to the ground [8, 14]. Various studies have reported burns with aberrant circuits at sites of patient uninsulated contact with the surgical table, electrocardiographic leads, motor-evoked potential monitoring electrodes, electroencephalogram electrode placement, and indwelling hardware [4, 8, 13, 15]. Therefore, great care should be taken to correctly connect the ESU and to make sure that it is functioning properly with no contact between the patient and any other grounding source so the current can travel within the circuit only.

Careful positioning in OR is teamwork, and patient safety must be assured by the whole team throughout the surgery [8, 10]. Patient positioned in prone is at increased risk of certain complications. Unique ophthalmic, neurologic, and myocutaneous complications could occur, as reported in the literature [11]. Knowledge of these possible complications is required to avoid them as they might inflect great harm and morbidity to the patient [12].

In our case, prone positioning had been carried out by the whole OR team, and despite the great efforts to properly position and protect the patient, the sidearm boards have fallen short to accommodate the whole fingers in the 90-90 degrees position of shoulder and elbow. This position made the fingers in a level that is slightly cephalic to the top of the head and closer to the anesthesia team. This site is away from the surgical field and not under the surgeon’s sight hence more vulnerable to unintended injuries. The completely metallic IV pole beside the patient used by the OR staff to hang the screen drape had come in contact with the patient’s fingers that were also hanging beyond the arm boards of the operating table. Movement of the IV pole or pulling of the drapes and subsequent contact must have occurred during surgery by either the surgical or anesthesia team and went unnoticed. Depending on the mechanism of ESU burn, burn sites can vary anywhere from head to lower extremities, but finger burns are unusual sites unless being the site of the aberrant circuit. The burn pattern in our patient is consistent with this mechanism as the dispersive pad was properly applied and functioning intraoperatively as per operative record, and there were no other burn sites postoperatively.

ESU burns have the potential to cause morbidity to patients and prolong hospital stay with the need for subsequent surgical interventions as debridement and skin grafting [7, 15]. A lawsuit is another aspect that lies a burden on patients and the health care system in these cases [16]. Our patient did not require further surgical intervention, but the hospital stay prolonged for five days more than the expected date of discharge. A high vigilance kept for any emerging new burn during her hospital stay as some have delayed appearance, as reported in the literature [7, 15]. The patient was treated conservatively in addition to serial follow-up visits and assessments until fully healed by the plastic surgery team and orthopedic spinal team. There was no functional deficit when the patient was assessed upon the last visit. However, there were small areas of soft tissue defects at burn sites similar in size to the original burns.

Attention should be paid while positioning patients in prone to avoid ESU injuries. The arms of the patient should have been fully accommodated in the sidearm boards in proper position with adequate padding. Unnecessary movements of the patient or the table should be completely avoided intraoperatively. IV poles should not be used to hang the drapes on for two reasons: firstly, it is not intended for this use. Secondly, any metallic object that could pose an alternative site for grounding should be kept away from the patient, especially if it is an object that is not insulated or stationary and could move intraoperatively near the patient’s body.

This pattern of burn in the prone position is not mentioned in the literature up to our knowledge. Patient safety is granted by whole OR staff, and such an injury is predictable, therefore preventable with a basic understanding of ESU principals and proper training for safe use, and with knowledge of its possible complications as well as for the prone position [9, 12, 16].

## Conclusions

Intraoperative burns can be caused due to a variety of reasons such as fires, chemical burns, and burns caused by electricity. ESU is an integral component of modern OR and has the potential to burn the patient or the surgeon. Newer designs reduce the complication of burns but do not eliminate it. Although being rare, these burns could inflect morbidity to the patient. Prone positioning is needed in spine surgeries and carries risks for injuries if not done properly as well as placing some parts of the body in more vulnerable positions. Intraoperative patient burn due to alternate current caused by a metallic object as IV pole contacting the patient’s hand in the prone position is possible and preventable. Patient safety is a shared responsibility of the OR team. ESU burns are predictable and could be avoided if the basic principles are taken into consideration by the whole OR team. Proper training in ESU use is invaluable and an essential part of surgeons’ armamentarium.
